# Pre-B acute lymphoblastic leukaemia recurrent fusion, *EP300-ZNF384*, is associated with a distinct gene expression

**DOI:** 10.1038/s41416-018-0022-0

**Published:** 2018-03-13

**Authors:** Barbara J. McClure, Susan L. Heatley, Chung H Kok, Teresa Sadras, Jiyuan An, Timothy P. Hughes, Richard B. Lock, David Yeung, Rosemary Sutton, Deborah L White

**Affiliations:** 1 Cancer Theme, South Australian Health and Medical Research Institute (SAHMRI), Adelaide, SA 5000 Australia; 2 School of Medicine, University of Adelaide, Adelaide, SA 5000 Australia; 3 Haematology Department, SA Pathology, Adelaide, SA 5000 Australia; 4 Children’s Cancer Institute, Lowy Cancer Research Centre, University of New South Wales, Sydney, NSW 2000 Australia; 5 School of Women’s and Children’s Health, University of New South Wales, Sydney, NSW 2000 Australia; 6 Australian Genomics Health Alliance (AGHA), Murdoch Children’s Research Institute, Parkville, VIC 3052 Australia; 7 School of Paediatrics, University of Adelaide, Adelaide, SA 5000 Australia

**Keywords:** Cancer genomics, Acute lymphocytic leukaemia

## Abstract

**Background:**

Zinc-finger protein 384 (*ZNF384*) fusions are an emerging subtype of precursor B-cell acute lymphoblastic leukaemia (pre-B-ALL) and here we further characterised their prevalence, survival outcomes and transcriptome.

**Methods:**

Bone marrow mononuclear cells from 274 *BCR-ABL1-*negative pre-B-ALL patients were immunophenotyped and transcriptome molecularly characterised. Transcriptomic data was analysed by principal component analysis and gene-set enrichment analysis to identify gene and pathway expression changes.

**Results:**

We exclusively detect E1A-associated protein p300 (*EP300*)*-ZNF384* in 5.7% of *BCR-ABL1*-negative adolescent/young adult (AYA)/adult pre-B-ALL patients. *EP300-ZNF384* patients do not appear to be a high-risk subgroup. Transcriptomic analysis revealed that *EP300-ZNF384* samples have a distinct gene expression profile that results in the up-regulation of Janus kinase/signal transducers and activators of transcription (JAK/STAT) and cell adhesion pathways and down-regulation of cell cycle and DNA repair pathways.

**Conclusions:**

Importantly, this report contributes to a better overview of the incidence of *EP300-ZNF384* patients and show that they have a distinct gene signature with concurrent up-regulation of JAK-STAT pathway, reduced expression of B-cell regulators and reduced DNA repair capacity.

## Introduction

Many genomic lesions in precursor B-cell acute lymphoblastic leukaemia (pre-B-ALL) are associated with alterations of cytokine receptors or their signalling pathway mediators, transcription factors or regulators of differentiation.^1,2^ These lesions have prognostic significance, for example, *ETV6-RUNX1* is associated with a relatively favourable outcome compared with poor-risk disease associated with *BCR-ABL*+ (Philadelphia-positive (Ph+)) or Ph-like ALL. Recently, next-generation sequencing studies have identified novel recurrent pre-B-ALL genomic lesions in genes such as *ABL1/2*, *JAK2*, *ZNF384*, *MEF2D* and *DUX4*,^3,4^ though the incidence and prognostic significance for some of these lesions are yet to be confirmed.

The E1A-associated protein p300 (*EP300*)*-ZNF384* fusion was initially reported as a recurrent fusion in 2015 with an incidence of 1.5% of paediatric B-ALL cases.^5^ Three additional studies have confirmed low incidence in paediatric populations (Supplementary Table 1), while adolescent and adult patients have a higher frequency.^3,6^ The *EP300* gene encodes a histone acetyltransferase (HAT), which influences transcription through chromatin remodelling and has tumour suppressor activity.^7^ The 3′ fusion partner zinc-finger protein 384 (*ZNF384*) encodes a C2H2-type protein that plays a role in transcription and nucleocytoplasmic transport. How *EP300-ZNF384* fusion protein expression alters gene transcription to promote leukaemic cell growth and survival is only beginning to emerge.^3,6,8^

In addition to *EP300*, multiple 5′ fusion partners of *ZNF384* have been reported in lymphoproliferative disorders including *TAF15,*^9,10^* ESWR1,*^9,10^ *CREBBP,*^6^* TCF3,*^6,9^* ARID1B,*^9^ *SYNRG*^3^ and *BMP2K,*^11^ implying that *ZNF384* is the predominant pathogenic lesion. *ZNF384* and the majority of its fusion partners are located within close proximity to the telomeres of their respective chromosome, making the identification of *ZNF384 *fusions difficult by conventional G-banding.^5,9^

Given the increasing importance of *ZNF384* as a recurrent genetic lesion in pre-B-ALL, we examined its frequency and prognostic significance in our cohort of 274 *BCR-ABL1*-negative pre-B-ALL patients on which we have transcriptomic sequencing data.

## Materials and methods

We studied 274 *BCR-ABL1*-negative pre-B-ALL patients (152 children, 54 adolescent/young adults (AYA, 16–39 years) and 68 adults) from the patient pool referred to us for Ph-like testing. Informed consent was obtained from each patient and the study was approved by the relevant institutional review board (Royal Adelaide Hospital Ethics Committee) and conducted in accordance with the Declaration of Helsinki. Transcriptomic sequencing data was generated using either Illumina HiSeq 2000 or NextSeq 500 platforms. Fusions and variants were identified (detail in Supplementary methods) and confirmed by reverse transcription polymerase chain reaction and Sanger sequencing. Samples were evaluated for a Ph-like signature by Taqman low-density array as previously described.^12^

For gene expression analysis, the raw fastq data was aligned by STAR aligner^13^ (version 2.4.2a) with two-pass method and described in full in the Supplementary methods, and only genes with false discovery rate (FDR) *p* < 0.05 were considered as statistically significant. Gene-set enrichment analysis (GSEA) was performed using Broad Institute GSEA software version 3.0 and Molecular Signature Database (MSigDB) version 5.2 (details are provided in Supplementary methods).

Five-year survival analysis of outcome data were estimated by Kaplan–Meier and log-rank test was used for significant difference, and we included Ph+ ALL cases for comparison. As part of our standard characterisation, immunophenotypic analysis was also performed for CD10, CD19 and CD34 expression.

## Results

**Fig. 1:**
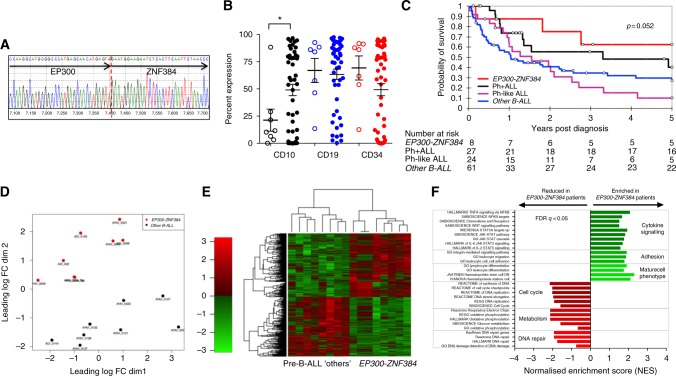
Pre-B-ALL expressing *EP300-ZNF384* MNC have reduced CD10 expression and a distinct gene signature, and patients have improved survival. **a** Sequence of the fusion breakpoint between *EP300* (at 22p13.2) and *ZNF384* (at 12p13). **b** Surface expression of CD10, CD19 and CD34 on AYA/adult MNC compared between pre-B-ALL containing *EP300-ZNF384* fusion (open circles) to those without detectable *EP300-ZNF384* fusions, pre-B-ALL ‘others’ (closed circles) *p = 0.046. Mean ± SEM is shown and analysed by unpaired *t-*test with two-tail test. **c** Kaplan–Meier analysis of percent overall survival from diagnosis for patients classified into different subtypes; *EP300-ZNF384*,* n* = 8 (red), B-ALL 'other', *n* = 61 (blue), *BCR-ABL1*, *n* = 27 (black) and Ph-like, *n* = 24 (purple). **d** Unsupervised clustering using multidimensional scaling (MDS) plots of log-CPM values based on dimensions 1 and 2 reveals differences in gene expression from 8 AYA/adult cases containing the *EP300-ZNF384* fusion (red) and 8 AYA/adult pre-B-ALL ‘other’ cases without detectable fusions (black) matched for age, initial white  cell count (WCC) and sex (outlined in Supplementary Table 2). The distances that display on the plot correspond to the average (root-mean-square) fold-change in log 2 scale for 500 genes with the most divergent between each pair of samples by default. An interactive MDS plot of this dataset can be found at the Supplementary Figure 1. **e** Differential gene expression in AYA/adult pre-B-ALL containing *EP300-ZNF384* fusion (red, *n* = 8) compared to those without detectable gene fusions (black, *n* = 8). Heatmap showing the top 100 genes with significant expression differences based on FDR *p* < 0.05. An interactive gene expression plot of this data set is available at https://github.com/chungkok/EP300_ZNF384.** f** Gene set enrichment profiling of transcriptomic sequencing data of *EP300-ZNF384* (*n* = 8) versus pre-B-ALL ‘other’ (*n* = 8) FDR *q* < 0.05. Statistical analysis was performed in GraphPad Prism (GraphPad Software Inc., La Jolla, CA, USA)

Transcriptomic sequencing revealed 7/122 (5.7%) AYA/adult and 2/152 (1.3%) children harboured the *EP300-ZNF384* fusion gene (patient details Table 1). One additional paediatric patient harboured a *TCF3-ZNF384* fusion. In 7/9 patients the *EP300-ZNF384* fusion was detected at diagnosis, and in the one patient where matched diagnosis and relapse samples were available, the fusion was detected in both. In the remaining two patients only the relapse sample was available for study. Patients with *EP300-ZNF384* fusions did not express a Ph-like gene signature. Eight of nine patients had identical break points at *EP300* exon 6 (22q13) and *ZNF384* exon 3 (12p13) (Fig. 1a), with the remaining patient having a *ZNF384* breakpoint in exon 2. The resulting truncation of *EP300* eliminates the HAT and bromodomain, which reportedly reduces HAT activity and binding of acetylated proteins.^8^ In contrast to other studies that report multiple upstream *ZNF384 *fusion partners,^3,6,8^ we identified predominantly *EP300-ZNF384* fusions in our cohort.

**Table 1:** Clinical findings and cytogenetic features of pre-B-ALL patients with *ZNF384* fusions

Patient	Sex	Event	Fusion	Age at event	Initial WBC 10^9^/ml	Initial CNS	Treatment	Karyotype	Variants	Outcome	Survival post Dx (years)
CH_A5049	M	Rel	*TCF3-ZNF384*	11.6		No data	ALLR3 therapy	47,XY,der(2)t(2;3)(p13;p21)t(2;7)(q33;q36),der(3)t(;3)(p13;p21),der(7)t(q33;q36),+8[6]/46,XY[18]		Alive	5.9
CH_A1498	M	Rel	*EP300-ZNF384*	16.6		No data	ALLR3 therapy	46,XY,t(2;17)(q13;q11.2),del(6)(?q15q23)[7]/46,XY[13]	RUNX1 E111K	Alive	17.1
CH_A2973	M	Rel	*EP300-ZNF384*	17.1	11	No data	ANZCHOG ALL8 therapy	46, XY, der (7) del (7) (q?)?inv (7) (q?) [12] /46, XY [7]	FLT3 Y589H	Died	4.4
AYAI_0004	M	Dx	*EP300-ZNF384*	16		No data	Study8	46,XY	KRAS T58I	Alive	6.9
AYAI_0004	M	Rel	*EP300-ZNF384*	23	2.58	Clear	Study8	45,X,-Y,t(1;12;6)(p36.1;q24.?3;p21),t(2;9)(q11.2;q1?2),del(19)(p13)[14]/46,XY[21]		Alive	
AYAII_0009	M	Dx	*EP300-ZNF384*	21	4.8	Clear	ALL6	45,XY,t(2;11)(p2?1;p15),del(6)(q13q2?3),inc[cp4]/46,XY[32].nuc.ish(TCF3x2)[200],(D4Z1,D10Z1,D17Z1)x2[200],(ABL,BCR)x2[200],(ABL,BCR)x2[200],(MLLx2)[200],(ETV6,RUNX1)x2[200]	NF1 Y1763F FLT3LG R216H	Alive	3.05
AYAII_0021	F	Dx	*EP300-ZNF384*	28	5.4	Clear	LALA	t(X;14)	NRAS Q61P PTK2B G414V ETV6 L201P EPOR N487S	Died	1.81
AYAII_0045	M	Dx	*EP300-ZNF384*	22	77.1	No data	ALSG	46,XY	CSFR1 N255I RUNX1 L29S	Died	11.63
ADI_0002	M	Dx	*EP300-ZNF384*	42		No data		46,XY	PTPN11 G60A		
ADI_0098	F	Dx	*EP300-ZNF384*	47	7.5	Clear	Hoeltzer	No data available	SH2B3 L6P EPOR P488S	Died	0.17
ADI_0159	F	Dx	*EP300-ZNF384*	43	25	No data		46,XX,t(13;14)(q14;q22)[11]/46,idem,t(12;22)(p13;q13)[4]/46,XX[45].ish 12p13(ETV6x2)[20],t(12;22)(WCP22+;WCP22−)[2/6].nuc ish(MYC,MLL,IGH)x2[100],(ASSx1,ABL1x1,BCRx2)[90/100]	NRAS G12C	Died	2.76

*pre-B-ALL* precursor B-cell acute lymphoblastic leukaemia, *LALA* Leucémies Aiguës Lymphoblastiques de l'Adulte, *ZNF384* zinc-finger protein 384, *WBC* white blood cell, *CNS* central nervous system, *Dx *  diagnosis, *Rel*  relapse

The patients harbouring *EP300-ZNF384* fusions had a median age of 24.5 years (range 4.4–47 years). In mononuclear cells (MNCs) of patients with *EP300-ZNF384* fusion (*n* = 8 available), the expression of CD10 was significantly lower when compared with other pre-B-ALL MNC (*n* = 48) (*p *= 0.046) (Fig. 1b). Surface expression of CD19 and CD34 was not significantly different between *EP300-ZNF384* (*n* = 7) and other pre-B-ALL groups (*p* = 0.7882 and *p* = 0.1916, respectively) (Fig. 1b). Of the five *EP300-ZNF384* patients where CD33 expression was available all had high expression.

Outcome data was available from 93 patients in our cohort (8 *EP300-ZNF384* patients, 24 Ph-like and 61 other pre-B-ALL). The 5-year survival of our cohort was 28.8%, with an average survival of 2.14 years. The notable poor survival seen in our AYA/adult pre-B-ALL cohort may reflect the inclusion of historical samples. Patients harbouring an *EP300-ZNF384* fusion had a 62.5% survival (*n* = 8) at 5 years. Outcomes for *EP300-ZNF384* patients compared favourably to other pre-B-ALL patients (27.2% 5-year survival; *n* = 61; *p* = 0.083) and Ph-like ALL patients^14^ (10.3% 5-year survival, *n* = 24; *p* = 0.019) (Fig. 1c), noting that four of the eight *EP300-ZNF384* patients were transplanted. For reference, we also included outcomes for 27 Ph+ ALL patients, the majority of whom were treated with tyrosine kinase inhibitor and chemotherapy, which had a 5-year survival of 40.3%.

Using transcriptomic sequencing, a variety of somatic variants were identified in *EP300-ZNF384* patients who are known to lead to missense alterations in transcription factors, cytokine receptors and signalling pathways in key cancer driver genes (Table 1). At least one variant was detected in each *EP300-ZNF384* case, including RAS mutations in three (33.3%) patients, NRAS G12C and Q61P, and KRAS T58I. Two (22.2%) patients had a RUNX1 mutation, E111K (*n* = 1) and L29S (*n* = 1). Mutations were also detected in the FLT3 signalling axis in two patients (22.2%), FLT3 Y589H (*n* = 1) and FLT3LG R216H (*n* = 1). Three patients (33.3%) had cytokine receptor mutations including EPOR P488S (*n* = 1) or N487S (*n* = 1) and CSF1R N255I (*n* = 1).

To identify how gene expression is altered when the *EP300-ZNF384* is expressed, we then compared the transcriptomic analysis of eight AYA/adult *EP300-ZNF384* cases to eight non-*EP300-ZNF384* cases matched for age, initial white  cell count and sex (patient details Supplementary Table 2). Unsupervised analysis using multidimensional scale (MDS) revealed that the *EP300-ZNF384* patient samples have a distinct gene expression profile compared to matched controls (Fig. 1d, Supplementary Figure 1). Gene expression analysis revealed differential expression of 984 genes (FDR *p* < 0.05) with similar numbers of genes up-regulated (476/984) or down-regulated (508/984). The heatmap (Fig. 1e) shows the differential gene expression based on the top 100 genes that discriminated between the two groups (Supplementary Table 3). Within the *EP300-ZNF384* cohort, up-regulation of *CLCF1* (*p* < 0.001), *CREB5* (*p* < 0.001), *STGALNAC2* (*p* < 0.001), *CD33* (*p* < 0.001) and *RUNX2* (*p* < 0.001) was observed and strong down-regulation of *OVCH2* (*p* < 0.001), *ARPP21* (*p* < 0.001), *NPY* (*p* < 0.001) and *BMP2* (*p* < 0.001). Expression of B-cell development regulators *PAX5* (*p* = 0.03), *IRF4* (*p* = 0.004) and *VPREB1* (*p* < 0.0001) were also significantly reduced in the *EP300-ZNF384* cohort. Altered regulation of myeloid reprogramming genes *GATA3*, *CEBPA* or *CEBPB* previously reported for this subgroup were not observed.^6^ Of note, the transcription factor, *KLF4*, which interacts with, and is acetylated by, EP300^15^ and negatively regulates PI3K signalling, was significantly down-regulated (log FC = −4.69, *p* < 0.001) in the *EP300-ZNF384* patients. KLF4 mediates TP53 action to regulate the G1–S phase transition following DNA damage. *TP53* expression was reduced by 1.98 fold in *EP300-ZNF384* patients compared to non-*EP300-ZNF384* patients (*n* = 8, *p* = 0.0141).

GSEA of our transcriptomic data showed that expression of *EP300-ZNF384* results in the up-regulation of genes related to Janus kinase/signal transducers and activators of transcription (JAK/STAT) signalling, leukocyte adhesion and differentiation pathways, whereas pathways related to cell cycle, oxidative phosphorylation and DNA repair are reduced (FDR *q* < 0.05) (Fig. 1f). GSEA analysis also revealed enrichment of genes containing motifs for LMO2 (FDR *q* = 0.016), RUNX1 (FDR *q* = 0.014) and GATA1 (FDR *q* = 0.012), all targets for ZNF384 interaction, and key regulators of haematopoiesis, and reduced expression from genes containing motifs for RB-1 (FDR *q* = 0.000).

The down-regulation of both *TP53* and *KLF4* in *EP300-ZNF384* samples may facilitate premature S phase entry prior to completion of DNA repair. This together with dampened EP300 acetylation function, a known modulator of TP53, may reduce genomic integrity^16^ and increase the potential for mutation acquisition in a setting of up-regulated JAK-STAT pathways and thus potentiate leukaemogenesis. *EP300-ZNF384* samples have reduced expression of both DNA repair reactome and DNA damage and repair pathways (Fig. 1f), suggesting that *EP300-ZNF384* expression contributes to higher genomic instability. Mutations or deletion of *TP53* are infrequent in pre-B-ALL but are common in many other tumours, and our transcriptomic data suggest that the down-regulation of DNA repair, relative to non-*EP300-ZNF384* pre-B-ALL, is an important feature of this cohort.

## Discussion

In contrast to other reports where *ZNF384 *fusions have multiple 5′ partners, we exclusively detected *EP300-ZNF384* fusions in 5.7% of *BCR-ABL1-*negative AYA/adult pre-B-ALL, making it one of the more prevalent recurrent lesions in this age group. Our *EP300-ZNF384* patients cluster in the AYA range and have reduced CD10 surface expression and up-regulated *CD33* expression in common with previous reports.^3,5^ The *EP300-ZNF384* subgroup showed improved outcome compared to other pre-B-ALL patients studied, and concurs with a Japanese cohort where *ZNF384* fusions have better survival outcomes than Ph-like ALL in AYA.^3^ This report contributes to a better overview of the incidence of *EP300-ZNF384* patients and the prognostic significance of *ZNF384* lesions deserves further study and validation. Importantly, we show here that the concurrent reduction in DNA repair capacity and activation of JAK-STAT pathways and B-cell regulators appear to be the hallmark features of the pre-B-ALL subtype expressing *EP300-ZNF384*.

## Electronic supplementary material

Supplementary Information
