# Supplementary material for: Pre-B acute lymphoblastic leukaemia recurrent fusion, EP300-ZNF384, is associated with a distinct gene expression
Source: Br J Cancer. 2018 Mar 13;118(7):1000–4. doi: 10.1038/s41416-018-0022-0 (PMC5931087; doi:10.1038/s41416-018-0022-0)
Supplement: Supplementary file 1 — Supplementary Information [file 41416_2018_22_MOESM1_ESM.docx]

| **Supplementary Table 1.** **Reported incidence of *EP300-ZNF384* fusions** | | |
| --- | --- | --- |
| **Incidence of *EP300-ZNF384*** | **Cohort Group** | **Reference** |
| 6/401 (1.5%) | Paediatric BCP-ALL, Japan | Gocho *et al*, 2015 |
| 1/195 (0.5%) | Paediatric BCP-ALL, Sweden | Lilljeborn *et al*, 2016 |
| 1/240 (0.4%) | Paediatric, Canada | Shago *et al*, 2016 |
| 3/92 (3.2%) | Paediatric, China | Liu *et al*, 2016 |
| 4/131 (3.1%) | Paediatric Ph-neg BCP-ALL, Japan | Yasuda *et al*, 2016 |
| 8/111 (7.2%) | Adolescent and young adult Ph-neg BCP-ALL, Japan | Yasuda *et al*, 2016 |
| 6/111 (5.4%) | Adult, China | Liu et al, 2016 |
| 2/152 (1.3%) | Children (<15yrs), Australia | This study |
| 7/122 (5.7%) | Adolescents and Adults, Australia | This study |

| **Supplementary Table 2. Clinical findings and cytogenetic features of control pre-B-ALL cohort for mRNA-Seq comparison.** | | | | | | | | |
| --- | --- | --- | --- | --- | --- | --- | --- | --- |
| **Patient** | **Sex** | **Event** | **Fusion** | **Age at event** | **Initial WBC 10^9/ml** | **Initial CNS** | **Treatment** | **Karyotype** |
|  |  |  |  |  |  |  |  |  |
| **AYAI_0020** | M | Dx | No fusion | 16 | 2.2 | Clear | LALA | 46, XY |
| **AYAII_0036** | F | Dx | No fusion | 37 | 5.7 | Clear | Hoeltzer |  |
| **AYAII_0101** | M | Dx | No fusion | 21 | 1.56 | Clear | LALA | t(Y;2)(q11.2;p1?1.2) |
| **AYAII_0107** | F | Dx | No fusion | 23 | 26.4 | No data |  | abnormal clone present with unidentified material replacing most of 4q, an extra 1q and monosomy 13 46,XX,add(4)(q21),-13,der(13)t(1;13)(q12;p11.2),+mar[6]/46,XX[14] |
| **AYAII_0127** | M | Dx | No fusion | 30 | 4.5 | Clear |  | 56,XY,+X,+4,+6,+10,,+14,+17,+18,+21,+21,+3mar[cp10]/46,XY[2] |
| **AYAII_0128** | F | Dx | No fusion | 30 | 9 | Clear | UKALL12 | 61-71,XXX,-X,+1,-3,-4,-4,+6,-7,-8,-9,+10,+11,+12, -13,-14,-15,-16,-17,-17,+19,-20,+21,+22,+mar1, +mar2,+mar3[cp6]/46,XX[14].nuc ish 9q34(ABLX2),22q11(BCRX4) |
| **AYAII_0132** | M | Dx | No fusion | 21 | 31.4 | Clear | ALL3 trial | 49,XY,+X,+21,+21[11]/47,XY,+12[8]/46,XY[1].ish 9q34(ABLx2),22q11(BCRx2) |
| **ADI_0144** | M | Dx | No fusion | 42 | 4.2 | Clear | Hyper-CVAD | 46,XY,der(16)t(1;16)(q?12:q1?2)[18]/46,XY[2].nuc ish(ABL1,BCR)x2[100],(MLLx2)[100] |

| **Supplementary Table 3. Top 100 deregulated genes** | | | |
| --- | --- | --- | --- |
| **Symbol** | **logFC** | **PValue** | **FDR** |
| *THRB* | 6.43 | 9.76E-12 | 1.34E-08 |
| *CLCF1* | 5.47 | 3.90E-13 | 1.32E-09 |
| *CREB5* | 5.23 | 8.19E-10 | 4.36E-07 |
| *GPR183* | 5.22 | 5.56E-12 | 9.39E-09 |
| *HBG1* | 4.97 | 7.92E-09 | 2.29E-06 |
| *HBG2* | 4.85 | 1.39E-06 | 1.90E-04 |
| *BTLA* | 4.78 | 2.63E-07 | 4.76E-05 |
| *IRGM* | 4.48 | 3.00E-08 | 7.07E-06 |
| *RUNX2* | 4.42 | 4.02E-16 | 2.04E-12 |
| *ST6GALNAC2* | 4.41 | 5.39E-17 | 5.46E-13 |
| *GCNT2* | 4.28 | 3.83E-10 | 2.28E-07 |
| *THBD* | 4.18 | 1.84E-06 | 2.43E-04 |
| *MYRIP* | 4.14 | 1.21E-08 | 3.30E-06 |
| *CD33* | 4.13 | 2.58E-11 | 2.61E-08 |
| *SNED1* | 4.03 | 3.01E-12 | 6.10E-09 |
| *SALL4* | 3.98 | 2.13E-06 | 2.76E-04 |
| *USP32P1* | 3.84 | 2.69E-08 | 6.48E-06 |
| *CXCL8* | 3.78 | 2.49E-06 | 2.93E-04 |
| *DLL1* | 3.64 | 9.29E-07 | 1.38E-04 |
| *MAF* | 3.63 | 2.24E-09 | 9.09E-07 |
| *FGL2* | 3.53 | 2.60E-06 | 3.02E-04 |
| *RIN3* | 3.48 | 1.32E-07 | 2.47E-05 |
| *REPS2* | 3.46 | 1.35E-10 | 1.05E-07 |
| *FRMD4A* | 3.46 | 2.41E-12 | 6.10E-09 |
| *SLC9A3R2* | 3.40 | 6.63E-09 | 2.05E-06 |
| *GSG2* | 3.36 | 3.41E-07 | 5.66E-05 |
| *MAP3K8* | 3.35 | 4.19E-06 | 4.29E-04 |
| *VIPR1* | 2.95 | 3.10E-06 | 3.31E-04 |
| *PXN* | 2.94 | 6.43E-08 | 1.33E-05 |
| *KIAA1671* | 2.89 | 1.28E-06 | 1.79E-04 |
| *DOK2* | 2.83 | 4.43E-08 | 9.55E-06 |
| *GAS2L1* | 2.71 | 1.73E-09 | 7.59E-07 |
| *LGALS3* | 2.53 | 9.76E-08 | 1.94E-05 |
| *LPPR2* | 2.53 | 1.30E-07 | 2.47E-05 |
| *SLC2A3* | 2.48 | 5.95E-09 | 2.01E-06 |
| *TNFRSF10A* | 2.45 | 2.26E-06 | 2.79E-04 |
| *FAS* | 2.42 | 3.96E-06 | 4.13E-04 |
| *ITGAE* | 2.37 | 3.11E-07 | 5.25E-05 |
| *MIR22HG* | 2.31 | 1.06E-06 | 1.56E-04 |
| *KLF7* | 2.23 | 2.26E-07 | 4.16E-05 |
| *CPEB4* | 2.23 | 2.17E-08 | 5.63E-06 |
| *SERPINB9* | 2.14 | 2.88E-06 | 3.14E-04 |
| *IRF5* | 2.09 | 2.37E-09 | 9.24E-07 |
| *RAP1GAP2* | 2.06 | 7.63E-10 | 4.29E-07 |
| *SLC35D2* | 2.02 | 7.15E-08 | 1.45E-05 |
| *GDPD5* | 1.95 | 2.06E-08 | 5.48E-06 |
| *PLXNA3* | 1.93 | 2.72E-06 | 3.09E-04 |
| *KLF3* | 1.82 | 1.45E-06 | 1.96E-04 |
| *TMBIM1* | 1.82 | 2.25E-06 | 2.79E-04 |
| *TMEM63B* | 1.77 | 2.67E-06 | 3.07E-04 |
| *ABCC1* | 1.71 | 2.85E-07 | 5.07E-05 |
| *NPEPL1* | 1.52 | 7.34E-07 | 1.13E-04 |
| *MYH11* | 1.50 | 4.16E-06 | 4.29E-04 |
| *SEMA4B* | 1.47 | 3.03E-07 | 5.21E-05 |
| *CD59* | 1.41 | 1.23E-06 | 1.76E-04 |
| *SEPHS1* | -1.39 | 4.45E-07 | 7.15E-05 |
| *GPX7* | -1.43 | 4.29E-06 | 4.34E-04 |
| *CDCA7L* | -1.45 | 1.24E-07 | 2.41E-05 |
| *EDEM1* | -1.46 | 2.47E-06 | 2.93E-04 |
| *AKR1A1* | -1.82 | 3.00E-07 | 5.21E-05 |
| *ABRACL* | -1.85 | 2.84E-06 | 3.14E-04 |
| *PARP1* | -1.85 | 6.36E-09 | 2.05E-06 |
| *GALNT2* | -1.88 | 2.44E-10 | 1.65E-07 |
| *PRKCE* | -1.89 | 2.24E-08 | 5.67E-06 |
| *SLAIN1* | -2.27 | 4.37E-08 | 9.55E-06 |
| *PDLIM1* | -2.58 | 4.43E-07 | 7.15E-05 |
| *TPD52* | -2.59 | 2.40E-08 | 5.92E-06 |
| *NREP* | -2.69 | 2.40E-06 | 2.90E-04 |
| *EPB41L2* | -2.81 | 5.70E-09 | 1.99E-06 |
| *LRIG1* | -2.85 | 1.85E-06 | 2.43E-04 |
| *PRDX1* | -2.85 | 2.76E-10 | 1.75E-07 |
| *AMOT* | -3.08 | 2.75E-06 | 3.09E-04 |
| *PHYH* | -3.09 | 4.11E-08 | 9.24E-06 |
| *CDCA7* | -3.19 | 4.63E-09 | 1.68E-06 |
| *CSRP2* | -3.20 | 1.54E-09 | 7.07E-07 |
| *NT5DC2* | -3.28 | 2.89E-06 | 3.14E-04 |
| *VPREB1* | -3.37 | 3.45E-06 | 3.64E-04 |
| *RIMS3* | -3.53 | 2.36E-06 | 2.88E-04 |
| *FGFR1* | -3.64 | 1.29E-06 | 1.79E-04 |
| *SPRED1* | -3.72 | 3.10E-06 | 3.31E-04 |
| *GPR125* | -3.78 | 4.10E-08 | 9.24E-06 |
| *APCDD1* | -4.35 | 1.22E-06 | 1.76E-04 |
| *GLDC* | -4.38 | 9.74E-10 | 4.70E-07 |
| *KLF4* | -4.69 | 8.13E-07 | 1.23E-04 |
| *TINAGL1* | -4.90 | 7.20E-07 | 1.12E-04 |
| *CTHRC1* | -4.90 | 3.03E-09 | 1.14E-06 |
| *MTCL1* | -4.92 | 5.87E-08 | 1.24E-05 |
| *COL27A1* | -5.00 | 9.69E-10 | 4.70E-07 |
| *APBB2* | -5.17 | 6.69E-09 | 2.05E-06 |
| *PHGDH* | -5.40 | 1.99E-09 | 8.41E-07 |
| *MYO1B* | -5.51 | 3.45E-11 | 3.17E-08 |
| *SDK1* | -5.60 | 2.18E-06 | 2.78E-04 |
| *CD1C* | -5.67 | 6.44E-07 | 1.02E-04 |
| *AGAP1* | -6.42 | 2.20E-06 | 2.78E-04 |
| *PARD3* | -6.74 | 3.76E-11 | 3.17E-08 |
| *CYB5R2* | -7.56 | 9.43E-09 | 2.65E-06 |
| *BMP2* | -8.34 | 7.00E-09 | 2.08E-06 |
| *NPY* | -9.40 | 2.16E-11 | 2.43E-08 |
| *ARPP21* | -9.62 | 2.14E-10 | 1.55E-07 |
| *OVCH2* | -10.32 | 1.06E-11 | 1.34E-08 |

**a**

**
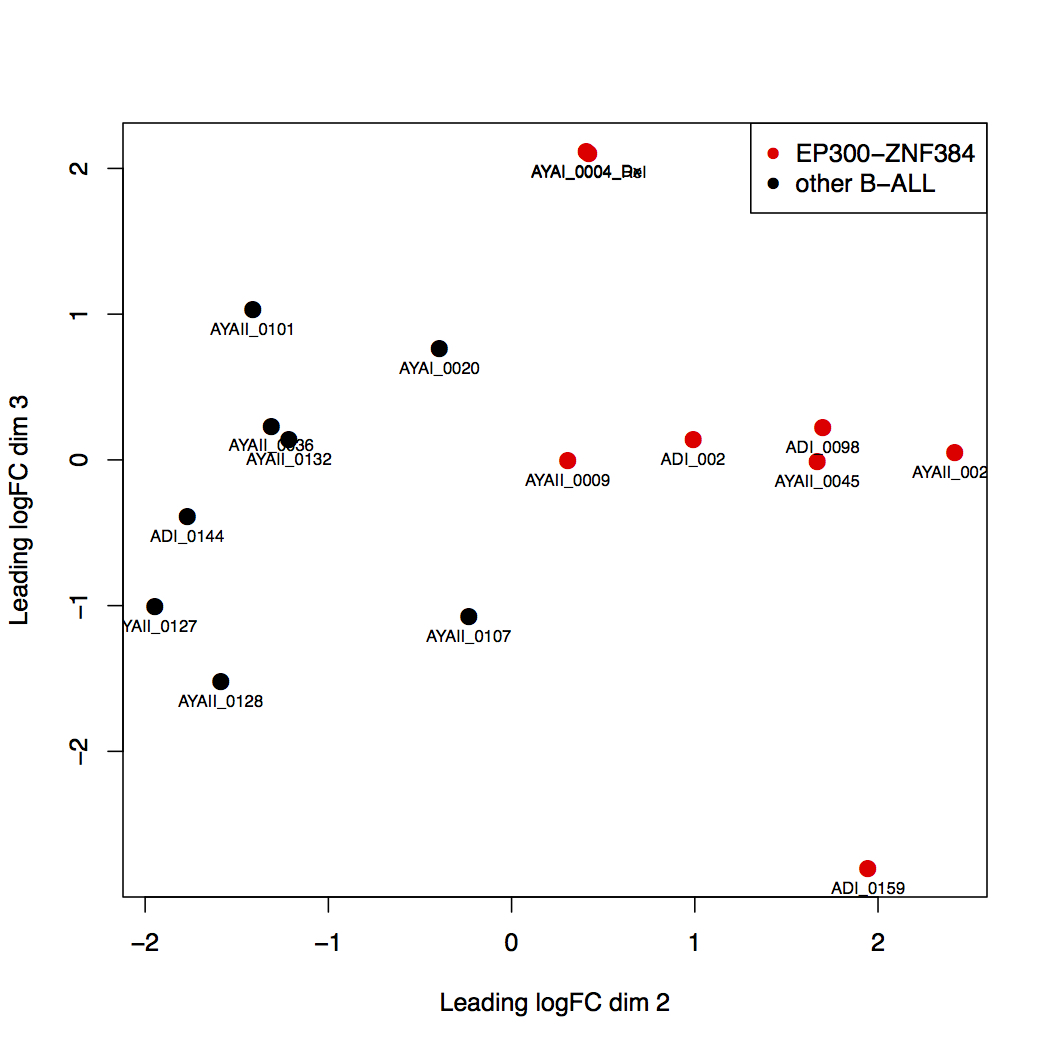
**

**b**

**
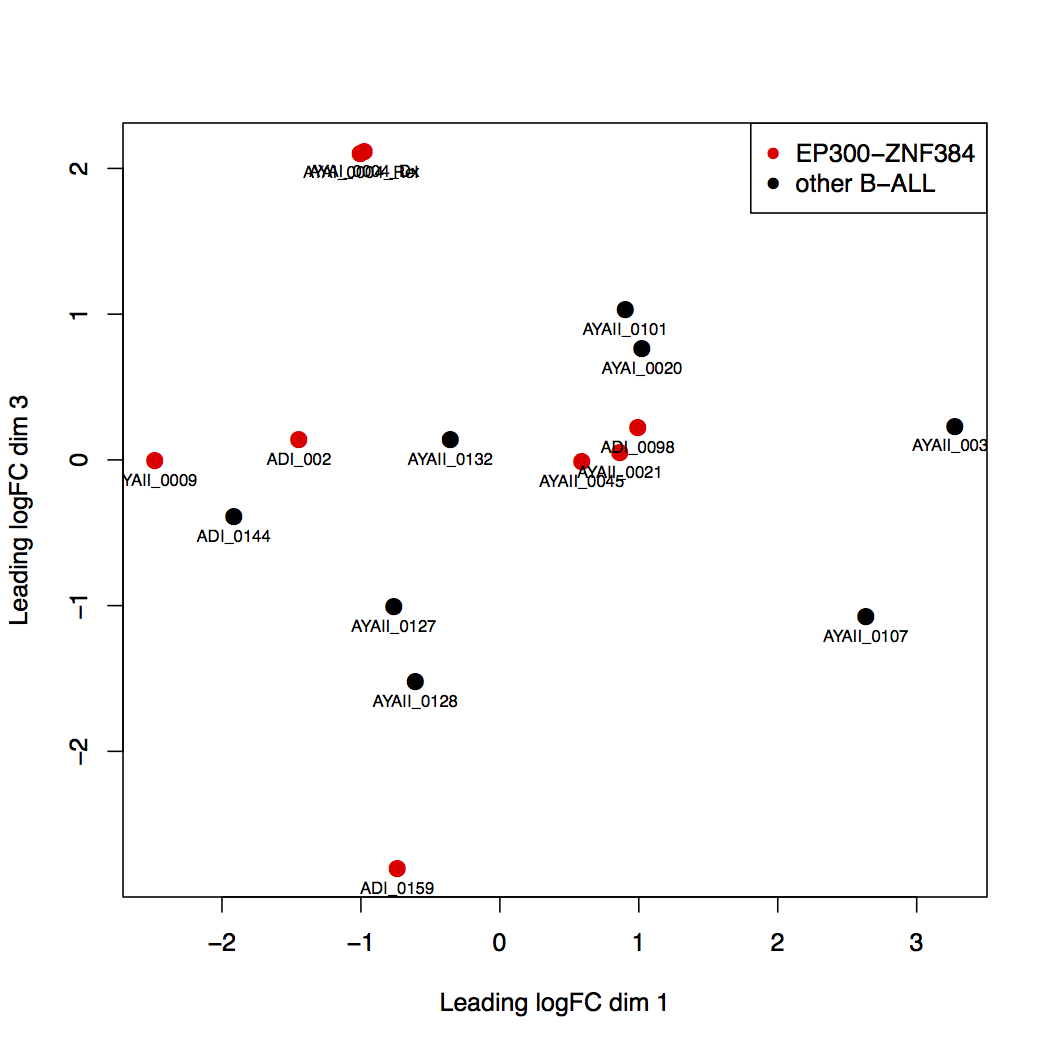
**

**Supplementary Figure 1**

**Supplementary Figure Legend**

**Supplementary Figure 1.** Unsupervised clustering using multidimensional scaling plots of log-CPM values based on dimensions 2 and 3 (a) and dimensions 1 and 3 (b) with samples labelled by their sample IDs and coloured by their sample groups, *EP300-ZNF384* (red) vs other B-ALL (black). The distances that display on the plot correspond to the average (root-mean-square) fold-change in log2 scale for 500 genes with the most divergent between each pair of samples by default. An interactive MDS plot of this dataset can be found at [https://github.com/chungkok/EP300_ZNF384](https://github.com/chungkok_EP300_ZNF384).

**Supplementary methods**

*Patient treatment*

Patients received intensive combination chemotherapy, and transplanted as per respective protocols. Ph+ ALL cases all received chemotherapy and tyrosine kinase inhibitors.

*Fusion and variant identification from Transcriptomic data*

Fusions were then identified using Fusion catcher and DeFuse software. Variants were called using GATK HaplotypeCaller (v3.4.46) (McKenna *et al*, 2010) and annotated by ANNOVAR software (2015-036-17), undergoing two further filtering steps, the first using SNPiR (Piskol *et al*, 2013) and the final filter being a set of 35 genes previously shown to associate with Ph-like ALL.

*Unsupervised clustering*

Unsupervised clustering was performed using multidimensional plot (MDS) as implemented in the bioconductor R package limma. The default setting for *plotMDS* function was used when performing the unsupervised clustering analysis. This function is a variation on the multidimensional scaling or principle coordinate plot. A distance is measured between each pair of samples using the root-mean-square deviation (Euclidean distance) based on log-CPM of the genes. The distances on the plot can be interpreted as leading log2 fold-change, which is the average (root-mean-square) log2 fold-change for the 500 genes most divergent between each pair of samples by default. For more details, please refer to *plotMDS* document as implemented in the limma package. An interactive MDS plot of this dataset was generated using Glimma package (Law *et al*, 2016,Ritchie *et al*, 2015).

*Gene set enrichment analysis*

For gene expression analysis, the raw fastq data was aligned by STAR aligner (Dobin *et al*, 2013) (version 2.4.2a) with 2-pass method. The resulting SAM file was then subjected to Picard (v1.136) processing steps (read group information, sorting, marking duplicates and indexing) and conversion to BAM format. The gene counts were performed using the featureCounts package (Liao *et al*, 2014) available from the Bioconductor in R statistical software. Differential gene expression was performed using edgeR package (Robinson *et al*, 2010), and only genes with FDR p<0.05 were considered as statistically significant. Gene-set enrichment analysis (GSEA) was performed using Broad Institute GSEA software version 3.0 and Molecular Signature Database (MSigDB) version 5.2. MSigDB is a collection of annotated gene sets for use with the GSEA software. Briefly, the pre-ranked gene list that ranked by edgeR was used to perform GSEA. The pre-ranked gene list was used to perform gene-set enrichment analysis against MSigDB hallmark gene sets (H), curated gene sets (C2), and gene ontology (GO) gene sets (C5), as well as SABiosciences complete list of pathway-focused gene sets (<http://sabiosciences.com/ArrayList.php)>. The default setting was used when performing the GSEA analysis. Only gene sets with FDR p<0.05 will be considered as statistically significant enriched in the gene list.

**Supplementary References**

Dobin, A, Davis, CA, Schlesinger, F, Drenkow, J, Zaleski, C, Jha, S, Batut, P, Chaisson, M, Gingeras, TR (2013) STAR: ultrafast universal RNA-seq aligner. *Bioinformatics,* 29**,** 15-21.

Law, CW, Alhamdoosh, M, Su, S, Smyth, GK, Ritchie, ME (2016) RNA-seq analysis is easy as 1-2-3 with limma, Glimma and edgeR. *F1000Res,* 5**,** 1408.

Liao, Y, Smyth, GK, Shi, W 2014. featureCounts: an efficient general purpose program for assigning sequence reads to genomic features. *Bioinformatics,* 30**,** 923-930.

McKenna, A, Hanna, M, Banks, E, Sivachenko, A, Cibulskis, K, Kernytsky, A, Garimella, K, Altshuler, D, Gabriel, S, Daly, M, DePristo, MA (2010) The Genome Analysis Toolkit: a MapReduce framework for analyzing next-generation DNA sequencing data. *Genome Res,* 20**,** 1297-1303.

Piskol, R, Ramaswami, G, Li, JB (2013) Reliable identification of genomic variants from RNA-seq data. *Am J Hum Genet,* 93**,** 641-651.

Ritchie, ME, Phipson, B, Wu, D, Hu, Y, Law, CW, Shi, W, Smyth, GK (2015) limma powers differential expression analyses for RNA-sequencing and microarray studies. *Nucleic Acids Res,* 43**,** e47.

Robinson, MD, McCarthy, DJ, Smyth, GK (2010) edgeR: a Bioconductor package for differential expression analysis of digital gene expression data. *Bioinformatics,* 26**,** 139-140.
